# Development, implementation, and evaluation of a belief-targeted non-face-to-face leisure-time physical activity promotion program for South Korean University students

**DOI:** 10.1186/s12889-026-28797-w

**Published:** 2026-08-03

**Authors:** Junhye Kwon, Joon-Ho Kang, Chung Gun Lee

**Affiliations:** 1 Department of Physical Education, College of Education, 71-1, Seoul National University, Seoul, 08826 South Korea; 2 Institute of Sport Science, Seoul National University, 1 Gwanak-ro, Gwanak-gu, Seoul, 08826 South Korea

**Keywords:** Leisure time physical activity, Physical activity promotion program, Belief-targeted intervention, Program evaluation, University students

## Abstract

**Background:**

Despite a sharp decline in physical activity levels among university students since the COVID-19 pandemic, physical activity programs for this population remain limited. Our previous research examined key beliefs influencing physical activity behavior among university students at a Korean university during the COVID-19 pandemic. Accordingly, this study aimed to develop, implement, and evaluate a non-face-to-face leisure-time physical activity (NFTF-LTPA) promotion program for university students, focusing on three beliefs—health, friends, and laziness—identified as key determinants of physical activity behavior in our previous study.

**Methods:**

Undergraduate students were newly recruited from the same university and departments (i.e., the College of Social Sciences and the School of Business) as our prior study. Participants were stratified and randomly assigned to intervention or control groups and a belief-targeted intervention was implemented for three months. Process evaluations were conducted at mid-, and post-intervention, and outcomes were assessed at pre-, mid-, and post-intervention. A one-month follow-up survey was also conducted after the intervention ended. A 2 × 4 repeated measures generalized linear model was conducted and subgroup analyses examined participants with high intervention adherence (PHIA) compared with controls.

**Results:**

Among the total analytic sample, significant group × time interaction effects at the α = 0.10 level were observed for outcome evaluation of behavioral beliefs (*F* = 2.70; *p* = 0.072; $\:{\eta\:}^{2}\:$= 0.05) and motivation to comply with normative beliefs (*F* = 2.54; *p* = 0.085; $\:{\eta\:}^{2}\:$= 0.05). However, no significant group × time interaction effects were observed for control beliefs, intention, or LTPA participation. In an exploratory subgroup analysis, a significant group × time interaction effect was observed for the control belief of laziness (*F* = 3.85; *p* = 0.031; $\:{\eta\:}^{2}$ = 0.18). Post hoc analyses indicated lower perceived laziness at the program midpoint in the PHIA group compared with the control group.

**Conclusions:**

Although the intervention produced changes in selected belief components among the total analytic sample, these changes did not translate into significant improvements in intention or behavior. Exploratory subgroup analyses suggested potential changes in control beliefs among participants with higher intervention adherence. These findings provide preliminary insights into the development, implementation, and evaluation of belief-targeted NFTF-LTPA promotion programs for university students.

**Trial registration:**

The trial was retrospectively registered at ClinicalTrials.gov (Identifier: NCT07423403; 19 February 2026).

**Supplementary Information:**

The online version contains supplementary material available at https://doi.org/10.1186/s12889-026-28797-w.

## Background

Physical inactivity is a serious public health concern worldwide, especially in the wake of COVID-19 [1, 2]. Beyond its direct health impacts, COVID-19 has substantially altered economic conditions, social interactions, and individual lifestyles on a global scale [3]. In particular, various measures such as the closure of sports facilities and restrictions on gatherings to prevent COVID-19 infection severely limited opportunities for physical activity (PA) participation [4, 5]. As a result, PA levels among university students worldwide have declined markedly since the COVID-19 pandemic. Empirical evidence further indicates that this decline was particularly pronounced during periods of lockdown, with a systematic review reporting significant reductions in PA among university students during COVID-19 confinements [5]. In addition, emerging evidence suggests that physical activity levels among university students have not consistently returned to pre-pandemic levels [6]. Moreover, low levels of physical activity among university students have been consistently observed even prior to the pandemic, highlighting this population as an important target for intervention.

Korean university students were no exception, with a notable decrease of approximately 23.4% in PA participation compared with the pre-pandemic period [7]. Factors such as the transition to online learning and restricted access to campus facilities may have limited students’ opportunities to benefit from on-campus PA resources [8]. Since they are still in a learning phase, there is a possibility for significant improvement in various health behaviors that can impact their later-life health [9]. Furthermore, it is widely known that PA patterns established in university have a long-term impact on adult PA habits [10, 11]. Therefore, it is essential to provide various policies and programs during this period to enable students to establish PA participation habits. However, there remains a shortage of PA programs for university students, particularly during COVID-19, and research interest in this area is still lacking in Korea.

Based on prior research identifying key beliefs influencing PA among Korean university students during the COVID-19 pandemic [12], this study aimed to develop, implement, and evaluate a leisure-time physical activity (LTPA) promotion program for this population. According to Park and Lee [12], salient beliefs such as ‘builds social relationships,’ ‘takes too much time,’ and ‘friends’ significantly predicted PA intention and behavior prior to the pandemic. During the pandemic, *“participating in physical activity improves my health”* (behavioral belief), *“my family members think that I should participate in physical activity”* (normative belief), and *“I think I am lazy to participate in physical activity”* (control belief) were significant predictors of PA intention and behavior although students’ average weekly PA time declined by half. This shift in salient beliefs likely reflects changes in the social and environmental context during the COVID-19 pandemic, in which restrictions on social interaction, increased health-related anxiety, and altered daily routines reshaped the cognitive determinants underlying PA intention and behavior [12]. These findings highlight the need for tailored PA interventions grounded in pandemic-specific determinants. The Theory of Planned Behavior (TPB) posits that behavioral, normative, and control beliefs shape individuals’ attitudes, subjective norms, and perceived behavioral control, which in turn influence behavioral intention and subsequent behavior [13]. In the present study, the selected beliefs were directly aligned with these constructs. ‘Health’ corresponds to behavioral beliefs, reflecting perceived outcomes of engaging in physical activity. ‘Friends’ corresponds to normative beliefs, representing perceived social expectations. ‘Laziness’ corresponds to control beliefs, reflecting perceived barriers to engaging in physical activity. Based on this framework, the intervention was designed to target these key beliefs to promote changes in intention and physical activity behavior. Accordingly, the present study adopted pandemic-specific behavioral, normative, and control beliefs as key targets for an LTPA promotion program designed to reflect the COVID-19 context. In the case of the normative belief ‘family members,’ it was deemed impractical for the researcher to directly intervene in family-related factors as a target belief. Therefore, ‘friends’ (“my friends think that I should participate in physical activity”) was set as the target belief to develop the program. This modification also reflects the university context and is consistent with TPB-based elicitation approaches that emphasize identifying salient referents within the target population [14, 15](Ajzen, 2002; Downs & Hausenblas, 2005). Thus, the substitution of ‘friends’ does not alter the theoretical structure of normative beliefs, but rather represents a context-specific application within the Theory of Planned Behavior framework. Importantly, participants in the present study were newly recruited from the same university and academic units as those included in the prior belief-identification study by Park and Lee [12]. This population continuity supports the application of previously identified salient beliefs to the development of the current LTPA intervention.

The COVID-19 pandemic has highlighted the importance of non-face-to-face (NFTF) physical activity interventions and accelerated their implementation across various settings. While such approaches are increasingly being adopted, physical activity interventions have traditionally relied on face-to-face delivery methods [16], and the evidence supporting NFTF models continues to develop [17, 18]. NFTF interventions offer greater efficiency in terms of time and cost, as they do not require direct physical interaction [17], making them particularly suitable during periods of infectious disease outbreaks such as COVID-19. Recent studies have also explored web-based interventions to promote physical activity among students. For instance, Paap et al. [19] demonstrated that a theory-driven web-based intervention can effectively enhance physical activity levels. Social media is considered one of the most effective channels for public health interventions in terms of its extensive reach and support for interaction and information [20–22]. Facebook may be well suited for health interventions due to its flexible content format and features such as pages and groups, which support the delivery of multi-component programs [23]. Furthermore, the COVID-19 pandemic has led to increased social media use, including longer daily Facebook usage among university students due to changes in communication patterns [24, 25]. Accordingly, Facebook represents a strategically appropriate platform for delivering physical activity interventions targeting young adults.

For a successful health promotion program, process evaluation is essential to determine whether intervention components were implemented as intended and to what extent [26, 27]. When significant behavioral changes occur, it is particularly important to identify which components contributed to these outcomes [28]. Process evaluation opens the “black box” of program implementation, enabling a deeper understanding of how specific components influenced outcomes [29, 30]. Although such analyses remain relatively limited, they provide critical insights for refining future interventions or identifying barriers when no behavioral change is observed [31, 32]. Therefore, this study incorporated a process evaluation to examine implementation fidelity and to better understand how intervention components contributed to behavioral outcomes.

To sum up, this study aims to develop, implement, and evaluate an NFTF-LTPA promotion program for university students, focusing on the three salient beliefs (i.e., health, friends, laziness) of physical activity that have been validated in the previous research conducted by Park and Lee [12].

Based on the Theory of Planned Behavior, the NFTF-LTPA promotion program was expected to produce positive changes in key beliefs, intention, and physical activity behavior.

Specifically, it was hypothesized that:

- H1. Participants in the intervention group would show greater improvements in behavioral, normative, and control beliefs compared to the control group.
- H2. Participants in the intervention group would demonstrate greater increases in intention to engage in physical activity compared to the control group.
- H3. Participants in the intervention group would show greater increases in leisure-time physical activity compared to the control group.

## Methods

### Study design

This study was designed as a stratified randomized controlled study to evaluate the effectiveness of a belief-targeted NFTF-LTPA intervention among university students. An a priori sample size calculation was conducted using G*Power (version 3.1.9.7). Based on a repeated measures design with two groups and four time points, a medium effect size (f = 0.25), a significance level of α = 0.05, a statistical power of 0.80, and an assumed correlation among repeated measures of 0.30, the required sample size was estimated to be 62 participants. The initial sample size of 77 participants met this requirement, accounting for potential attrition. 

### Participants

This study was conducted as a follow-up to a previous study by Park and Lee [12], and participants were undergraduate students recruited from the same department at same university located in Seoul, South Korea. Consistent with the previous study, students were recruited from the College of Social Sciences and the School of Business. Participants were recruited via group email invitations linking to a pre-intervention questionnaire assessing LTPA-related beliefs, intentions, and behaviors. Those who agreed to participate provided their mobile phone number for program-related communication.

To prevent imbalances in LTPA time and gender distribution between groups [33], a stratified randomization procedure was applied. Participants first completed a baseline survey assessing LTPA participation, gender, and psychological variables. Participants were stratified based on baseline LTPA levels (≥ 150 min/week vs. <150 min/week), gender, and key psychological variables (control beliefs and perceived power), which were exhibited relatively high variability at baseline. Within each stratum, participants were randomly assigned to either the intervention or control group. Blinding was not feasible due to the nature of the intervention, as participants were aware of their group allocation through active engagement in the Facebook-based program.

This study was approved by the Institutional Review Board (IRB) of XXX University and written informed consent was obtained from all participants prior to participation. A formal data monitoring committee was not established because the study involved a low-risk behavioral intervention. The trial was retrospectively registered at ClinicalTrials.gov (Identifier: NCT07423403; 19 February 2026). 

### Intervention

The LTPA program, specifically designed based on the aforementioned three salient beliefs (health, friends, and laziness), was developed using Facebook pages and groups, incorporating various strategies to increase LTPA participation rates. The program content was delivered through posts, peer interactions, and activity-based tasks, with participants encouraged to engage by viewing content, interacting with others, and uploading activity-related materials. In line with previous evidence indicating that participation in web-based PA programs tends to decline after three months [34], the program was conducted from May 8 to August 6, 2023. The intervention group was divided into five subgroups (6–7 members each), following Rote et al.’s recommendation of 5–9 participants per subgroup [35]. These subgroups were created to facilitate peer interaction and group-based activities during the intervention and were not analyzed as independent analytic subgroups. Meanwhile, the control group did not receive any program-related content or intervention materials during the intervention period. The LTPA program consisted of six intervention arms as follows:

1. Providing LTPA related information

Information on the utilization and accessibility of sports facilities, commonly identified as environmental factors affecting PA participation [36–38], was shared on the page (e.g., campus trails). The informational posts targeted three beliefs: health, friends, and laziness.

2) Goal setting

**Fig. 1:**
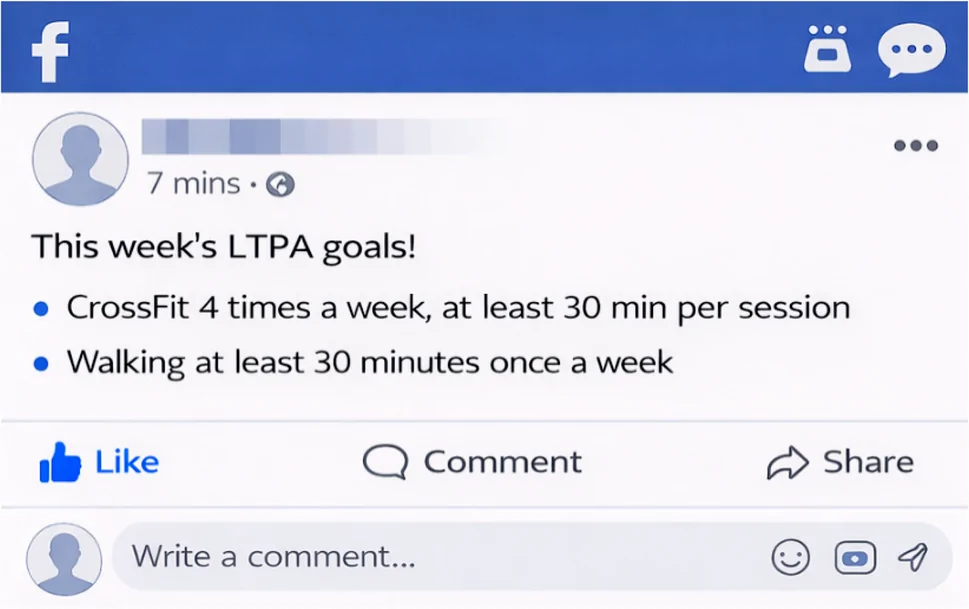
Example of participants posting LTPA weekly goals (English translation)

Laziness is primarily described as an emotion caused by a lack of motivation. Goal setting, self-discipline, and reward-based interventions have been shown to be an effective strategy for improving motivation to participate in PA [15, 39, 40]. To reduce perceived laziness toward LTPA participation, participants were instructed to post weekly LTPA goals (frequency, duration, and activity type) in their groups every Monday (see Fig. 1).

3) Verification of the LTPA participation

**Fig. 2:**
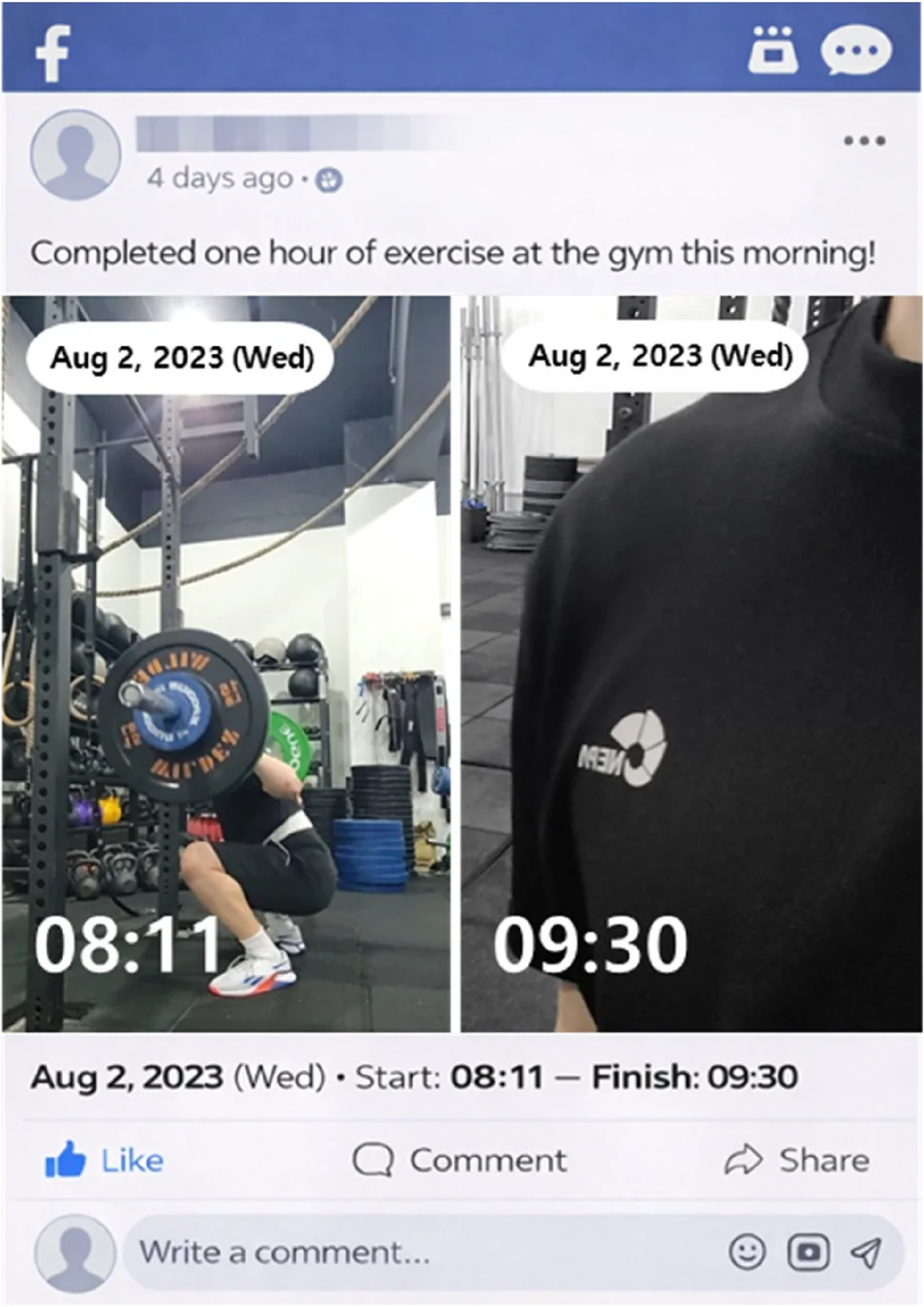
Example of LTPA participation verification images (English translation)

This study assured individuals’ actual LTPA participation by having them verify their activities within each group. The verification method is to post photos that included individual’s appearance (excluding face), the LTPA location/facility, date, and the starting and finishing time of the activity, providing a degree of behavioral validation beyond self-report (see Fig. 2).

4) Group incentive

Regardless of personal goals, when more than 70% of members in a group achieved at least 150 min of LTPA per week, all members of the group received an incentive worth approximately $5, as verified by the researcher. The group-based incentive was applied only within the five intervention subgroups and was not provided to the control group. Goal setting, verification of LTPA participation, and group incentives were implemented as interconnected interventions, and these three strategies may help reduce feelings of laziness toward LTPA participation. The financial incentive was also included as a strategy to promote participant engagement and sustained participation in both program activities and LTPA throughout the intervention period.

5) Normative message

**Fig. 3:**
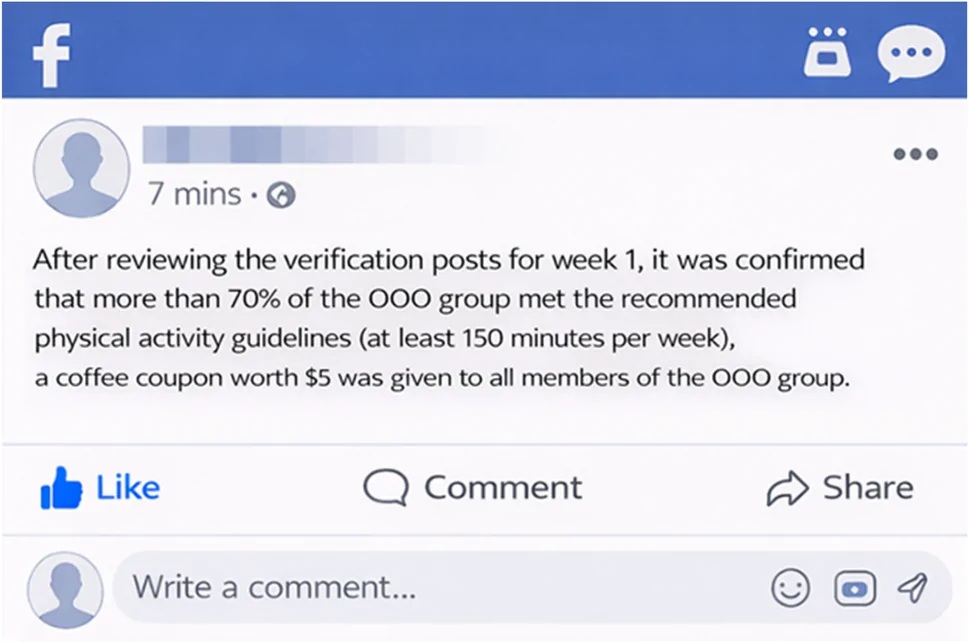
Example of normative messages (English Translation)

Based on evidence that peer-related social norms influence university students’ PA [41–43], normative messages were delivered to promote positive changes in normative beliefs regarding friends and to enhance LTPA-related intentions and behaviors. Specifically, following verification of group posts, the researcher shared the percentage of each group meeting PA recommendations and posted information on incentive-eligible groups, thereby allowing all participants to view weekly LTPA results (see Fig. 3).

6) Role model

**Fig. 4:**
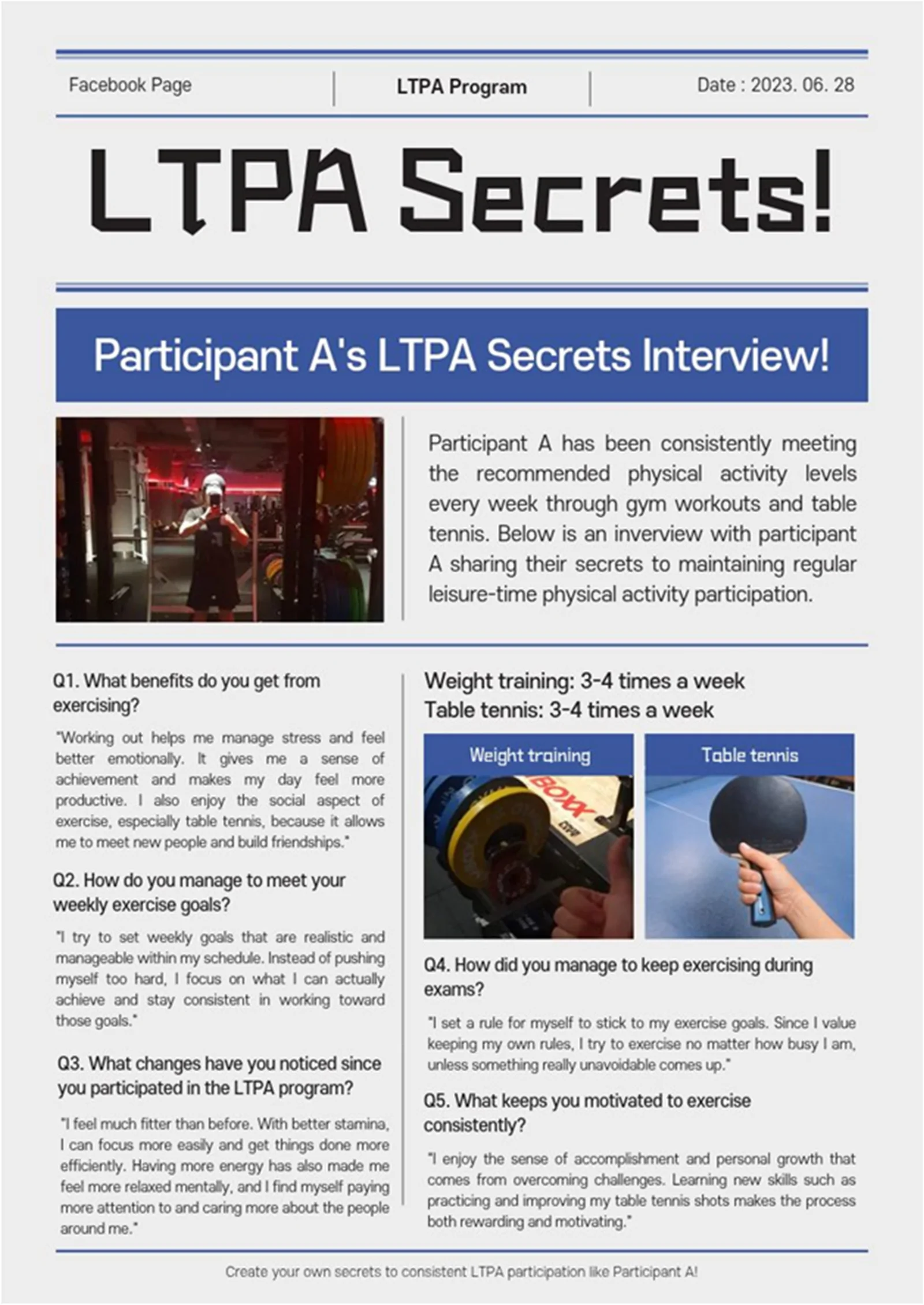
Example of a role model interview (English translation)

A role model interview was included to target behavioral belief ‘health’ and normative belief ‘friends’. Two participants who engaged in at least 150 min of LTPA per week during the initial six weeks of the program were selected as role models. They were interviewed via Facebook Messenger regarding program-related changes and personal strategies for LTPA participation, and their responses were shared within each group and on the Facebook page (see Fig. 4).

The intervention was designed to influence key belief constructs underlying physical activity behavior, with the aim of promoting changes in intention and subsequent behavior. Informational posts were used to address all three beliefs (health, friends, and laziness), while additional components were intended to primarily reinforce specific constructs. Health-related messages were expected to influence behavioral beliefs, peer interaction and normative messaging were aimed at strengthening normative beliefs (friends), and goal-setting and activity verification strategies were used to address perceived barriers related to laziness (control beliefs). Although some components involved group-based elements and may have influenced multiple beliefs simultaneously, their primary functions were conceptually distinguished, with normative strategies focusing on social influence and control-related strategies addressing motivational barriers. Through these mechanisms, the intervention was expected to influence intention and subsequent physical activity behavior. 

### Data collection

**Fig. 5:**
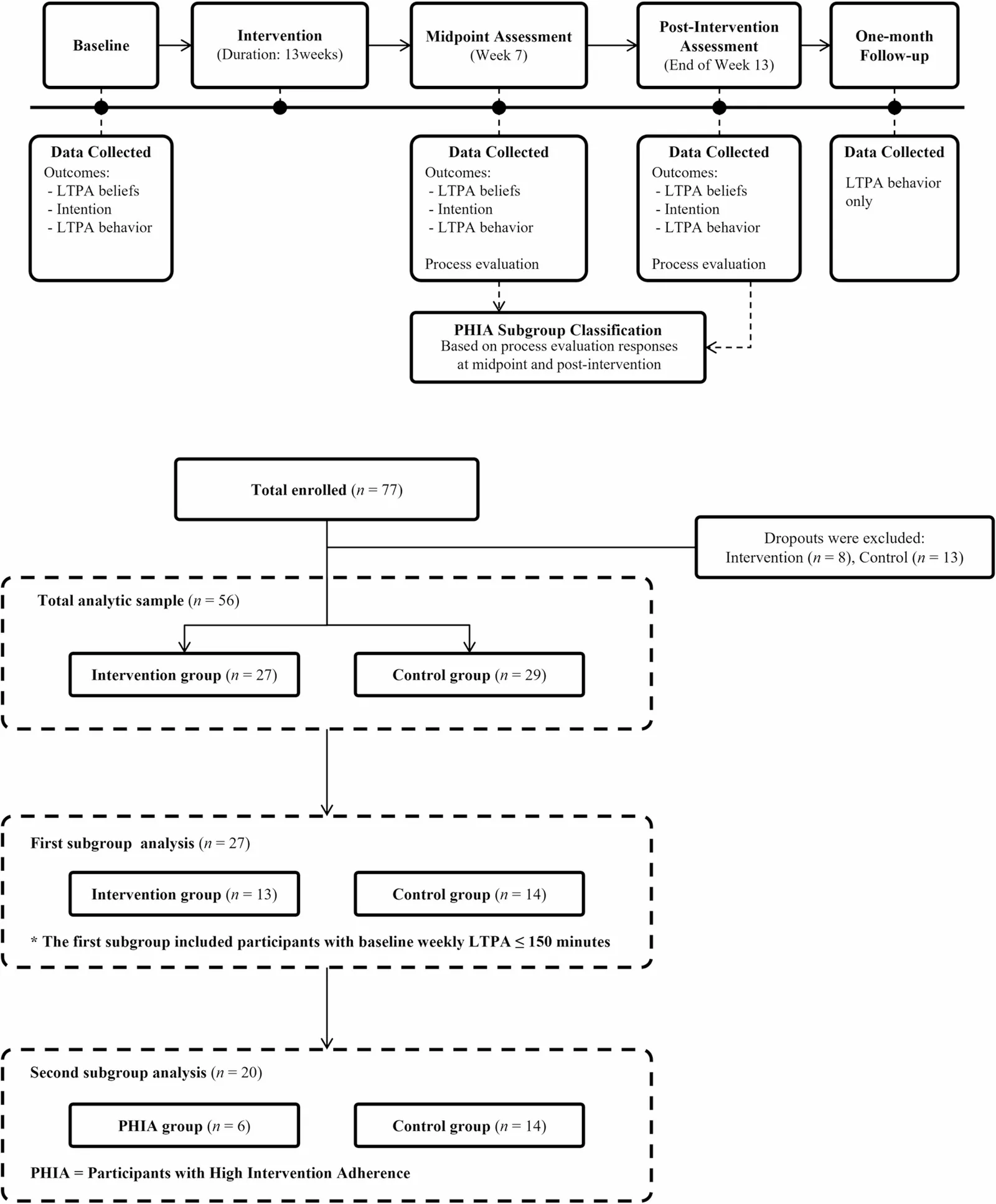
Timeline of the NFTF-LTPA program and data collection procedures

Since all procedures of this study were conducted remotely, both the process and outcome evaluations of the LTPA program were carried out using a Google online survey. Process evaluations were conducted at mid-, and post-intervention, and outcomes were assessed at pre-, mid-, and post-intervention. A one-month follow-up survey was also conducted after the intervention ended (See Fig. 5).

Process evaluation was conducted to assess participants’ engagement with the intervention, adherence to program activities, and the fidelity of implementation. Specifically, engagement was evaluated based on participants’ exposure to and interaction with intervention components (e.g., viewing posts, setting goals, and logging activities), while adherence reflected participation in program-related activities and receipt of intervention content. In addition, implementation fidelity was assessed by evaluators in terms of the appropriateness of content delivery, participant understanding, and overall engagement in group activities. Detailed items are provided in the supplementary tables.

### Process evaluation by participants

Process evaluation items were included in the main survey administered at the midpoint and post-intervention. Items used at the program midpoint and end are presented in Supplementary Tables S1 and S2, respectively, with three additional items included at post-intervention. All process evaluation items were assessed using dichotomous (Yes/No) responses and were adapted from previous studies [44–46]. These items were administered only to the intervention group. In addition, an open-ended question was included to collect participants’ qualitative feedback on their experiences with the program.

### Process evaluation by evaluators

To objectively evaluate LTPA program, a process evaluation was conducted at the midpoint of the intervention by three evaluators. The evaluation panel consisted of one professor with expertise in health behavior and promotion and two doctoral candidates in the same field. All intervention content shared via Facebook was provided to the evaluators, who completed a 10-item process evaluation survey (see Supplementary Table S3) [44–46].

### Outcome evaluation

Outcome variables included behavioral, normative, and control beliefs, intention, and LTPA behavior. The outcome measures were developed based on the Theory of Planned Behavior framework and adapted from a previously published study [12], in which the measurements were adopted from validated sources and further developed following established guidelines. The items used in the present study were derived from salient beliefs identified through an elicitation study conducted in the previous study. Behavioral, normative, and control beliefs, as well as intentions regarding LTPA participation [47], were evaluated using a nine-point Likert scale (response options ranged from − 4 = extremely unlikely to 4 = extremely likely) at pre, mid, and post intervention stages (See Table 1). The control belief item assessed perceived laziness (i.e., whether participation in LTPA would feel bothersome), conceptualized as a perceived barrier that may hinder engagement in LTPA. The intention measure consisted of three TPB-based items adapted from previous research [47], which were selected based on the TPB framework identifying intention as the most proximal determinant of behavior. The three items of intention were computed to represent an average score for intention to engage in LTPA in the next month. Internal consistency for intention was examined using Cronbach’s alpha at each assessment point. The two items of LTPA participation were analyzed by multiplying frequency and duration to derive the weekly average LTPA participation time over the past month as a single variable. A one-month follow-up was conducted solely to measure the level of LTPA participation.

**Table 1:** Questions of behavioral, normative, and control beliefs, intention and behavior regarding LTPA participation

Outcomes	Questions
BB	“Do you think participating in LTPA can improve your health?”
OE	“Do you think improving health is good or not good?”
NB	“Do you think your friends would agree or disagree with your participation in LTPA?”
MC	“Are you willing to follow your friends’ opinions or thoughts?”
CB	“Do you think participating in LTPA would feel bothersome?”
PP	“Do you think the laziness makes LTPA participation difficult or hinders it, or does it help or make it easier?”
Intention	“Do you intend to participate in LTPA in the next month?”
	“Will you make an effort to participate in LTPA in the next month?”
	“Do you plan to engage in LTPA in the next month?”
LTPA	“How many days per week did you engage in LTPA during the past month?”
	“How many minutes per day did you spend on LTPA participation?”

Perceived power items were assessed using a bipolar nine-point Likert scale ranging from -4 (extremely difficult) to +4 (extremely easy)

*Abbreviation*: *BB* Behavioral Belief, *OE* Outcome Evaluation, *NB* Normative Belief, *MC* Motivation to Comply, *CB* Control Belief, *PP* Perceived Power

### Data analysis

The results of process evaluation were presented as mean (standard deviation, SD) and percentage. A 2 × 4 repeated measures generalized linear model (GLM) was used to examine the effects of group (intervention vs. control), time (pre, mid, post, and one-month follow-up), and their interaction (group × time) on participants’ perceptions, intention, and behavior related to LTPA. The main effect of time reflects overall changes across measurement points, the main effect of group represents overall differences between the intervention and control groups across all time points, and the interaction effect (group × time) was interpreted as the differential change over time between groups, representing the effect of the intervention. To further examine group differences over time, effect sizes were calculated using partial $\:{\eta\:}^{2}$ and Cohen’s d values. For repeated measures data, the assumption of sphericity was assessed prior to analysis, and appropriate corrections were applied when necessary [48]. In cases of significant interaction effects, post hoc tests were conducted. The significance level was set at α = 0.10, considering the sample size [49], to reduce the risk of Type II error; however, this approach may increase the risk of Type I error and therefore requires cautious interpretation.

Based on the World Health Organization’s PA guidelines [50], two separate subgroup analyses were conducted. Because the intervention was designed to promote LTPA participation, participants who did not meet PA recommendations at baseline were expected to have greater opportunity for behavioral change than participants who were already sufficiently active. Therefore, the first subgroup included participants with baseline weekly LTPA ≤ 150 min, and comparisons of LTPA perceptions, intentions, and behaviors were conducted between the intervention and control groups. The second subgroup assessed program effectiveness by comparing these outcomes between participants with high intervention adherence (PHIA) in the intervention group and the control group, all of whom did not meet PA recommendations at baseline. The PHIA subgroup analysis was conducted as an exploratory analysis to examine whether intervention adherence was associated with differential outcomes among participants who were initially inactive. The PHIA subgroup was defined as participants who demonstrated high engagement with the intervention procedures and support protocol. Specifically, participants who responded “yes” to all core process evaluation items related to active engagement with the NFTF-LTPA intervention procedures during the midpoint and post-intervention process evaluations were classified into the PHIA subgroup, excluding supplementary content engagement items (items 2, 7, and 8 in Supplementary Table S2). These core items assessed active participation in the intervention, including exposure to program content, interaction within the Facebook group, weekly goal posting, exercise logging, and receipt of program-related messages. Only participants with complete data were included in the analyses. All analyses were conducted using IBM SPSS 26.0 and SAS 9.4 statistical programs.

## Results

### Characteristics of participants

A total of 77 participants were initially enrolled and randomly assigned to the intervention (*n* = 35) or control group (*n* = 42). The retention rates through the one-month follow-up were 77.1% for the intervention group and 69.0% for the control group. Therefore, the total analytic sample included 56 participants (*n* = intervention: 27, control: 29) who completed all four assessment points (see Fig. 5). Among the total analytic sample, there was a higher proportion of female students compared to male counterparts (male: 41.4%, female: 53.6% shown in Supplementary Table S4). The results of the baseline survey revealed that the LTPA participation time of the intervention and control groups was 190.74 and 201.03 min, respectively.

### Process evaluation

A total of 31 participants completed the process evaluation at the midpoint of the LTPA intervention. Most participants reported viewing LTPA-related posts, with high engagement in viewing posts shared by other participants, while goal-setting and activity logging were reported by approximately two-thirds of participants (presented in Supplementary Table S1).

At post-intervention, overall adherence to the intervention decreased compared with the midpoint evaluation (presented in Supplementary Table S2). Although the proportion of participants who checked page posts remained high, fewer participants reported thoroughly reading the content. Engagement in group activities, including goal-setting and verification posts, also declined. In addition, approximately half of the participants reported checking role model-related posts, with fewer indicating that they read these posts in detail.

The results of the process evaluation conducted by the evaluators are presented in Supplementary Table S3. The evaluators showed the lowest agreement regarding participants’ enjoyment of page posts and active engagement in group activities (Mean = 2.3, SD = 0.58; Mean = 2.0, SD = 2.0). Moderate agreement was observed for items assessing careful reading of page posts and attention to textual content (Mean = 3.0, SD = 1.0). For the item assessing participants’ understanding of group activities, one evaluator strongly agreed and two moderately agreed (Mean = 3.3, SD = 0.58).

### Sphericity test

The results of Mauchly’s sphericity test indicated that the assumption of equal covariance matrices across all levels within the group was violated, as the p-value was lower than the α = 0.10 level set in this study (W = 0.05056189, *p* < 0.10). Therefore, this study used the corrected estimates based on the Huynh-Feldt Epsilon (H-F).

### Outcome evaluation

The intention scale demonstrated high internal consistency reliability across baseline, mid-intervention, and post-intervention assessments (Cronbach’s α = 0.90, 0.97, and 0.96, respectively).

Table 2 shows the results of differences in LTPA-related beliefs, intention, and behavior among the total analytic sample. Significant group × time interaction effects at the α = 0.10 level were observed for outcome evaluation of behavioral beliefs (*F* = 2.70; *p* = 0.072; $\:{\eta\:}^{2}\:$= 0.05) and motivation to comply with normative beliefs (*F* = 2.54; *p* = 0.085; $\:{\eta\:}^{2}\:$= 0.05). However, no significant group × time interaction effects were observed for control beliefs, intention, or LTPA participation.

**Table 2:** Differences in LTPA beliefs, intention, and behavior between the intervention and control groups (*N* = 56)

Outcomes	Intervention Group (*n* = 27)				Control Group (*n* = 29)				Group *F*	Time *F*	GT *F*
	Pre	Mid	Post	1-month Follow-up	Pre	Mid	Post	1-month Follow-up			
Mean (SD)											
LTPA time	190.74 (153.77)	166.15 (162.73)	187.22 (189.35)	207.41 (169.36)	201.03 (208.09)	149.48 (150.79)	171.72 (143.06)	138.62 (114.64)	0.47	0.91	1.00
Intention	2.38 (1.34)	2.25 (1.57)	2.32 (1.62)		1.60 (1.81)	2.01 (2.10)	2.53 (1.67)		0.55	1.77	2.22
BB	3.19 (0.79)	3.00 (1.49)	3.15 (1.03)		2.93 (1.16)	3.00 (1.16)	3.21 (0.94)		0.08	0.60	0.50
OE	3.59 (0.69)	3.81 (0.48)	3.78 (0.42)		3.72 (0.96)	3.45 (1.33)	3.79 (0.49)		0.18	1.08	2.70^*^
NB	2.48 (1.37)	3.00 (1.24)	2.96 (1.06)		2.14 (2.17)	2.69 (1.44)	2.62 (1.05)		1.45	3.16^*^	0.00
MC	2.33 (1.57)	2.48 (1.65)	2.04 (2.08)		1.14 (2.23)	1.76 (2.06)	2.07 (1.56)		2.59	1.12	2.54^*^
CB	1.00 (2.18)	0.78 (2.34)	1.30 (2.18)		0.48 (1.88)	0.97 (1.86)	0.76 (1.90)		0.35	0.88	1.84
PP	1.63 (2.10)	2.19 (1.47)	2.11 (1.69)		0.76 (2.17)	1.28 (1.98)	1.83 (1.79)		3.42^*^	3.63^**^	0.71

*Abbreviation*: *BB* Behavioral Belief, *OE* Outcome Evaluation, *NB* Normative Belief, *MC* Motivation to Comply, *CB* Control Belief, *PP* Perceived Power, *SD* Standard Deviation, *GT* Group˟Time

^**^p < 0.05, ^*^p < 0.10

Table 3 presents the results of differences in LTPA-related beliefs, intention, and behavior among participants who did not meet the 150 min/week physical activity guideline at baseline. A significant time effect was observed for LTPA participation (*F* = 2.46; *p* = 0.089; $\:{\eta\:}^{2}\:$= 0.09), whereas no significant group or group × time interaction effects were found.

**Table 3:** Differences in LTPA beliefs, intention, and behavior among the first sub-groups (*N* = 27)

Outcomes	Intervention Group (*n* = 13)				Control Group (*n* = 14)				Group F	Time F	GT F
	Pre	Mid	Post	1-month Follow-up	Pre	Mid	Post	1-month Follow-up			
Mean (SD)											
LTPA time	78.46 (45.98)	124.31 (143.83)	144.23 (203.26)	146.92 (166.95)	68.57 (50.67)	81.79 (79.85)	128.57 (134.90)	98.21 (82.13)	0.62	2.46^*^	0.31
Intention	2.31 (1.47)	1.95 (2.00)	2.03 (1.98)		0.95 (1.86)	1.38 (2.21)	2.14 (1.89)		1.05	0.85	1.80
BB	3.15 (0.80)	3.15 (0.99)	3.31 (0.85)		2.93 (0.83)	2.93 (1.21)	3.29 (1.14)		0.27	1.10	0.17
OE	3.69 (0.63)	3.92 (0.28)	3.85 (0.38)		3.93 (0.27)	3.50 (1.09)	3.86 (0.53)		0.14	0.45	2.44
NB	2.46 (1.51)	3.08 (1.12)	3.31 (0.75)		2.50 (2.18)	2.57 (1.50)	2.50 (1.29)		1.16	0.82	0.75
MC	2.23 (1.79)	2.38 (1.45)	2.00 (1.96)		1.29 (2.13)	1.71 (2.05)	1.93 (1.64)		1.07	0.28	0.62
CB	1.62 (2.06)	1.15 (1.82)	1.77 (1.83)		1.50 (1.45)	1.71 (1.54)	1.29 (1.64)		0.00	0.11	1.82
PP	2.38 (1.45)	2.15 (1.68)	2.77 (1.17)		1.64 (2.10)	1.57 (1.83)	1.93 (1.73)		1.83	1.17	0.08

*Abbreviation*: *BB *Behavioral Belief, *OE* Outcome Evaluation, *NB* Normative Belief, *MC* Motivation to Comply, *CB* Control Belief, *PP* Perceived Power, *SD* Standard Deviation, *GT* Group˟Time

^**^p < 0.05, ^*^p < 0.10

The results of differences in LTPA-related beliefs, intention, and behavior between the PHIA intervention group and the control group are presented in Table 4. No significant main or group × time interaction effects were observed for behavioral beliefs. A significant time effect was observed for motivation to comply with normative beliefs (*F* = 2.64; *p* = 0.088; $\:{\eta\:}^{2}\:$= 0.13), indicating changes across the measurement periods.

**Table 4:** Differences in LTPA beliefs, intention, and behavior among the second sub-groups (*N* = 20)

Outcomes	PHIA Group (*n* = 6)				Control Group (*n* = 14)				Group F	Time F	GT F
	Pre	Mid	Post	1-month Follow-up	Pre	Mid	Post	1-month Follow-up			
Mean (SD)											
LTPA time	101.67 (24.01)	211.67 (174.63)	257.50 (261.11)	211.67 (219.49)	68.57 (50.67)	81.79 (79.85)	128.57 (134.90)	98.21 (82.13)	4.67^**^	3.53^**^	0.94
Intention	2.89 (0.98)	3.28 (0.85)	3.39 (0.95)		0.95 (1.86)	1.38 (2.21)	2.14 (1.89)		5.57^**^	2.11	0.44
BB	3.17 (0.98)	3.33 (0.82)	3.50 (0.55)		2.93 (0.83)	2.93 (1.21)	3.29 (1.14)		0.59	0.85	0.07
OE	3.33 (0.82)	3.83 (0.41)	3.83 (0.41)		3.93 (0.27)	3.50 (1.09)	3.86 (0.53)		0.19	0.56	2.34
NB	2.33 (1.51)	3.33 (1.03)	3.33 (0.82)		2.50 (2.18)	2.57 (1.50)	2.50 (1.29)		0.78	0.81	0.71
MC	1.83 (2.48)	3.17 (1.33)	3.17 (0.98)		1.29 (2.13)	1.71 (2.05)	1.93 (1.64)		2.10	2.64^*^	0.50
CB	1.17 (1.94)^a^	0.17 (1.72)^b^	1.33 (1.86)		1.50 (1.45)	1.71 (1.54)	1.29 (1.64)		0.72	1.08	3.85^**^
PP	2.33 (1.51)	1.33 (1.97)	2.17 (1.47)		1.64 (2.10)	1.57 (1.83)	1.93 (1.73)		0.10	1.14	0.57

*Abbreviation*: *BB* Behavioral Belief, *OE* Outcome Evaluation, *NB* Normative Belief, *MC* Motivation to Comply, *CB * Control Belief, *PP* Perceived Power, *SD* Standard Deviation, *GT* Group˟Time

^**^p < 0.05, ^*^p < 0.10

^a^ pre-mid, ^b^ mid-post

**Fig. 6:**
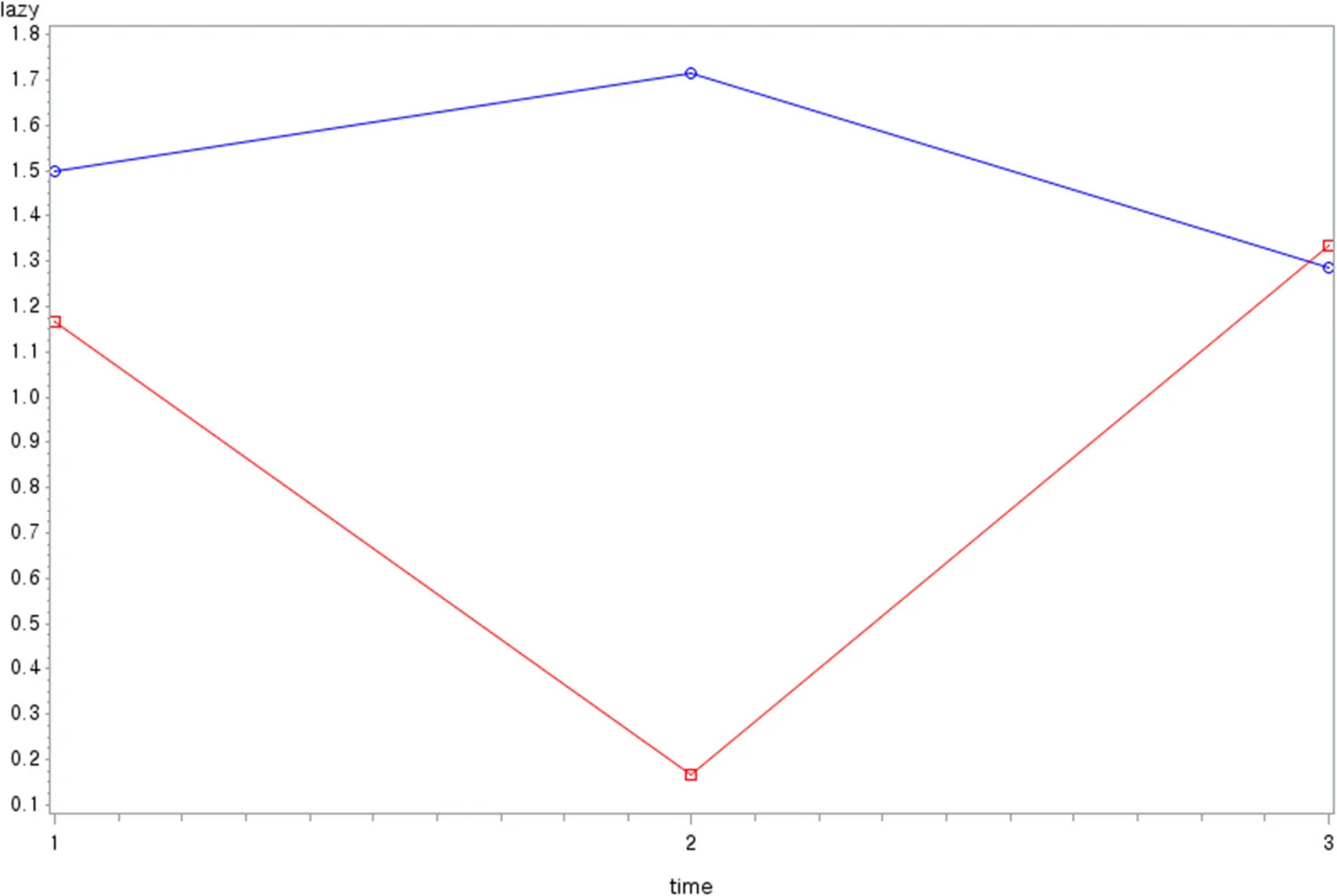
Group × Time interaction in control belief related to perceived barriers to LTPA participation in the second subgroups

For control beliefs, a significant group × time interaction effect was observed, with a large effect size (*F* = 3.85; *p* = 0.031; $\:{\eta\:}^{2\:}$= 0.18). Post-hoc analyses indicated that participants in the PHIA intervention group reported significantly fewer perceived barriers to LTPA participation at the midpoint of the program compared with the control group (*F* = 6.07; *p* = 0.024; *d* = 0.90). Although control belief scores in the PHIA group increased slightly between the midpoint and post-intervention assessments (see Fig. 6), the midpoint comparison demonstrated a substantially larger effect size than the corresponding change observed in the control group at the end of the program (*d* = -0.02).

For LTPA intention, a significant group effect was observed (*F* = 5.57; *p* = 0.030; $\:{\eta\:}^{2}\:$= 0.24), indicating higher overall intention scores in the PHIA intervention group across the measurement periods compared with the control group.

For LTPA participation, significant group (*F* = 4.67; *p* = 0.044; $\:{\eta\:}^{2}\:$= 0.21) and time (*F* = 3.53; *p* = 0.038; $\:{\eta\:}^{2}\:$= 0.16) effects were observed; however, no significant group × time interaction effect was found. Although descriptively higher LTPA participation levels were observed in the PHIA intervention group across the measurement periods, these differences were not statistically supported by a significant interaction effect.

## Discussion

The present study aimed to develop, implement, and evaluate a belief-targeted NFTF-LTPA promotion program for university students based on previously identified salient beliefs related to LTPA participation. It was expected that participation in the program would be associated with positive changes in LTPA-related beliefs, intention, and behavior. Significant group × time interaction effects were observed for outcome evaluation of behavioral beliefs and motivation to comply with normative beliefs among the total analytic sample. However, no significant interaction effects were observed for control beliefs, intention, or LTPA participation. Overall, although the intervention produced changes in selected belief components, these changes did not translate into significant improvements in intention or behavior. Therefore, H1 was only partially supported, whereas H2 and H3 were not supported. Similarly, among participants who did not meet PA recommendations at baseline, no significant group × time interaction effects were observed for LTPA-related beliefs, intention, and behavior. Although the primary hypotheses were not entirely supported, the second exploratory subgroup analysis provided additional insight into potential differences in outcomes according to intervention adherence. In particular, significant changes were observed for control beliefs among participants with higher intervention adherence, whereas changes in behavioral beliefs, normative beliefs, intention, and LTPA participation remained limited. However, these findings should be interpreted cautiously because the subgroup analysis was exploratory in nature and involved relatively small sample sizes. Furthermore, the observed effects may reflect not only the NFTF delivery format but also the financial incentive and engagement-promoting components incorporated into the intervention. In this second subgroup analysis, behavioral beliefs remained unchanged despite the intervention. One possible explanation is that health-related behavioral beliefs may have already been relatively well established among these participants prior to the intervention, limiting the potential for further change. Previous research has suggested that stronger or more established attitudes tend to be more resistant to change [51]. Taken together, the subgroup findings may suggest that different TPB-related beliefs vary in their responsiveness to intervention efforts, highlighting the need for more differentiated strategies in future theory-based intervention design. The PHIA group showed higher intention scores than the control group across the assessment periods. However, given the exploratory nature of this subgroup analysis, caution is warranted when interpreting the relationship between intention and subsequent behavior. Descriptively, the LTPA participation rate of the PHIA group more than doubled at the midpoint of the program compared with baseline, and higher LTPA participation levels were observed relative to the control group. However, these observations should be interpreted cautiously because no group × time interaction effect was found for LTPA participation. Moreover, the PHIA group exhibited an early increase in motivation to comply with friends that was sustained after the program midpoint, whereas the control group showed only a modest and gradual increase over time. This may suggest that interactions with other group members were associated with positive changes in beliefs, and the continuous provision of normative messages may have indirectly influenced students’ perceptions. These findings are broadly consistent with previous research employing subgroup analyses to examine the effects of Facebook-based PA interventions [52]. The present findings can also be interpreted in relation to underlying psychological processes. Previous web-based intervention studies [19], have shown that theory-driven interventions can improve motivational factors related to physical activity. Similarly, theory-based interventions may influence psychological factors related to physical activity, although the present study provided limited evidence for corresponding changes in intention and behavior. However, our findings should be interpreted with caution, particularly given the small sample size of the PHIA group and the limited number of statistically significant effects.

Moreover, reductions in the control belief of laziness were observed alongside changes in physical activity behavior at the midpoint of the program. In terms of effect size, the substantial decrease in perceived laziness among the PHIA group may have been associated with the group-based activities embedded in the intervention. Students set weekly LTPA participation goals and worked to achieve them, resulting in increased engagement in PA during their leisure time. Additionally, they tended to set their personal LTPA goals to exceed 150 min in line with the incentive conditions. White et al. [40] proposed goal-setting and reward-based strategies as an approach to reduce perceptions of laziness regarding control beliefs and to increase motivation for PA. In this context, the group activities of this program may have supported participants’ efforts toward goal achievement. Moreover, by using the incentive strategy, participants were indirectly encouraged to have a shared goal within the group, which may have stimulated the group dynamics of students and contributed to greater engagement in program activities. The interaction and sense of fellowship formed among group members to achieve the same goal is likely to have a positive impact on PA behavior change [53]. These findings are generally consistent with previous digital and social media-based physical activity interventions emphasizing the importance of social support, peer interaction, and group dynamics in promoting physical activity participation. In particular, the shared goals and interaction among participants observed in the present study may have contributed to sustained motivation and engagement throughout the intervention period. Estabrooks et al. [54] reviewed the effectiveness of PA interventions based on a group dynamics approach and reported that, regardless of the combination of intervention strategies, the group dynamics approach was generally effective. These findings also suggest that future research may also benefit from applying more systematic classifications of motivational behaviors, such as those proposed by Ahmadi et al. [55], to develop and evaluate more targeted physical activity interventions for university students.

According to the process evaluation results, the level of intervention adherence decreased after the program midpoint. This is likely because the program midpoint overlapped with the final exam period, and university students likely prioritized their academic work over participating in the program. In fact, the participants highlighted their lack of active participation due to exam preparations in their responses to the last question of the midpoint process evaluation, saying, ‘I couldn’t participate due to the exam period, but I plan to rejoin starting today,’ ‘There was too much to do during the exam period, so I couldn’t participate in the program,’ and ‘If the program were conducted outside the exam period, participation rates would be higher.’ These results align with previous research showing that university students face difficulties in engaging in PA during the final exam period [56]. The decline in participant engagement during the exam period may also help explain the reduced intervention effects observed over time. In particular, lower levels of participation in program-related activities, such as goal-setting and activity verification, may have reduced continued exposure to intervention components designed to influence beliefs and physical activity behavior. These findings may suggest that sustained engagement is an important consideration in the implementation of NFTF-based physical activity interventions.

As reflected by the incentive-based engagement patterns, study participants were more actively involved in group activities during the first 6 weeks, before the final exam period and the end of the semester. A study measuring the effectiveness of health behavior interventions targeting university students reported that interventions lasting within one semester are more effective in changing specific health behaviors than those lasting over one semester [8]. Although the duration of this program was shorter than a full semester, it started in the middle of the semester and continued through the vacation period after the semester ended, which is likely why the intervention adherence dropped in the later stages. Therefore, future research should take into account the characteristics of university students and their academic schedules when planning such programs.

This study has several strengths worth highlighting. First, this study conducted a process evaluation to assess the effectiveness of the program, which provided useful information for interpreting the results. In particular, the process evaluation by the evaluators, which was based on the opinions of a third party excluding the researchers and participants, is significant in that it was utilized as relatively objective evaluation data. Moreover, these evaluation results will be used as an important framework by future researchers in the field when developing similar programs. Second, this study developed a NFTF-LTPA promotion program based on the changed PA beliefs of university students following the outbreak of COVID-19. While numerous studies have been conducted both domestically and internationally on COVID-19 and university students’ participation in PA [5], there has been little research on interventions based on PA beliefs that consider the social atmosphere and environmental circumstances of that period. This study is meaningful in that it implemented a tailored behavior change strategy to promote belief changes among students from the same university, in line with previous studies that examined PA beliefs. Furthermore, since this study developed the program while considering that COVID-19 had not yet ended, the program was implemented in an online format. Therefore, it will serve as valuable data for future situations involving similar diseases and infections. Third, the provision of incentives through exercise verification distinguishes this study from previous PA interventions that used Facebook groups. Students who set LTPA goals and participated in group-based activities reported lower perceptions of laziness and higher levels of LTPA participation during the intervention period. These observations suggest that group-based approaches may represent a promising strategy for supporting changes in university students’ perceptions and behaviors regarding LTPA. They will serve as valuable resources for developing PA-related programs and policy elements for university students in the future.

Nevertheless, it is important to acknowledge the following limitations. First, the data for this study were collected through self-administered questionnaires, which may have led to social desirability and recall bias. Although efforts were made to minimize these biases through exercise verification, participants could not be objectively verified in all cases, and it cannot be ruled out that some LTPA participation occurred without verification. In addition, blinding of participants was not feasible due to the interactive nature of the Facebook-based intervention, which may have increased the possibility of expectancy or response bias. Second, although the measures used in this study were based on previously validated TPB instruments, several modifications to the original scale format were made to better fit the context of the intervention. Therefore, it is possible that these adaptations may have influenced participants’ response patterns to some extent. Future research should improve the accuracy of the findings by using more objective measurement methods, such as heart rate monitors and accelerometers. Third, in this study, two participants who most actively participated in LTPA during the first 6 weeks of the program were selected as a role model to foster positive changes in LTPA beliefs. This program was developed based on PA beliefs from the previous study conducted with university students, and therefore, influencers [57] or celebrities, who have been shown to effectively influence PA behaviors, were not considered as role model subjects. Future research using social media interventions should consider implementing various role models that have been shown to effectively influence PA intention and behaviors in university students, not only peer friends. Fourth, this study did not assess the feasibility or reach of the intervention through metrics such as ‘’likes’’, ‘’comments’’, or the number of logins. Future research should use more objective assessment tools to evaluate the feasibility and reach of intervention tools. Fifth, the small sample size of the PHIA subgroup may limit statistical power and the stability of the estimates. Although stratified randomization was applied, baseline differences in intention were observed between groups. Therefore, findings related to intention should be interpreted with caution, particularly given the exploratory nature of the subgroup analyses and the small sample size. Finally, because group-based incentives were provided only to the intervention group, it is difficult to distinguish the effects of the intervention itself from the potential influence of incentives and competitive group dynamics. Accordingly, any observed changes should not be attributed solely to the NFTF delivery format. Therefore, caution is needed when interpreting the effectiveness of the intervention and generalizing the findings to real-world settings where such incentives may not be available.

## Conclusion

The primary analyses partially supported the study hypotheses. Significant group × time interaction effects were observed for outcome evaluation of behavioral beliefs and motivation to comply with normative beliefs, whereas no significant effects were found for control beliefs, intention, or behavior. The second exploratory subgroup analysis further suggested that participants with higher intervention adherence showed significant changes in control beliefs related to perceived barriers to LTPA participation. These findings should be interpreted cautiously given the exploratory nature of the subgroup analyses, the small sample size, and the limited number of significant effects. Nevertheless, the present study provides preliminary insights into the development, implementation, and evaluation of belief-targeted LTPA promotion programs for university students in situations involving infectious disease-related restrictions.

## Supplementary Information

Supplementary Material 1.

## Data Availability

The datasets generated and/or analyzed during the current study are not publicly available due to the terms of consent/assent to which the participants agreed, but are available from the corresponding author upon reasonable request.

## Abbreviations

NFTF: Non-Face-to-Face
LTPA: Leisure-Time Physical Activity
PHIA: Participants with High Intervention Adherence
PA: Physical Activity
IRB: Institutional Review Board
SD: Standard Deviation
GLM: Generalized Linear Model
