# Supplementary material for: Development, implementation, and evaluation of a belief-targeted non-face-to-face leisure-time physical activity promotion program for South Korean University students
Source: BMC Public Health. 2026 Aug 3;26:3130. doi: 10.1186/s12889-026-28797-w (PMC13721259; doi:10.1186/s12889-026-28797-w)
Supplement: Supplementary file 1 [file 12889_2026_28797_MOESM1_ESM.docx]

Table S1. Responses to questions of mid process evaluation asking whether participants experienced each intervention (N = 31).

| Questions | Yes (%) |
| --- | --- |
| 1. Did you see the LTPA-related posts on the Facebook page? | 30 (96.8) |
| 2. Did you see other participants' posts in the Facebook group? | 27 (87.1) |
| 3. Did you post your weekly LTPA goals in the Facebook group? | 20 (64.5) |
| 4. Did you log your exercise to achieve your weekly LTPA goals in the Facebook group? | 20 (64.5) |
| 5. Did you receive messages related to the NFTF-LTPA promotion program? | 31 (100.0) |

Table S2. Responses to questions of post process evaluation asking whether participants experienced each intervention (N = 30).

| Questions | Yes (%) |
| --- | --- |
| 1. Did you see the LTPA-related posts on the Facebook page? | 28 (93.3) |
| 2. Did you read the LTPA-related posts on the Facebook page in detail? | 18 (60.0) |
| 3. Did you see other participants' posts in the Facebook group? | 90 (76.7) |
| 4. Did you post your weekly LTPA goals in the Facebook group? | 17 (56.7) |
| 5. Did you log your exercise to achieve your weekly LTPA goals in the Facebook group? | 18 (60.0) |
| 6. Did you receive messages related to the NFTF-LTPA promotion program? | 29 (96.7) |
| 7. Did you see the 'LTPA participation tips' posts by OOO and OOO in the Facebook page and group? | 16 (53.3) |
| 8. Did you read the 'LTPA participation tips' posts by OOO and OOO in detail? | 12 (40.0) |

Table S3. Responses to questions of process evaluation asking whether interventions were implemented as planned (N = 3).

| Item | Mean (SD) |
| --- | --- |
| *Facebook Page* |  |
| 1. Was the number of posts on the page appropriate? | 4.00 (0.00) |
| 2. Did the participants carefully read the posts provided on the page? | 3.00 (1.00) |
| 3. Did the participants like the posts on the page? | 2.30 (0.58) |
| *Facebook Groups* |  |
| 1. Did the researcher provide a detailed explanation about group activities related to LTPA participation? | 4.00 (0.00) |
| 2. Did the researcher provide feedback on the participants' posts? | 4.00 (0.00) |
| 3. Was the number of participants in the group appropriate? | 4.00 (0.00) |
| 4. Did the participants fully understand group activities? | 3.30 (0.58) |
| 5. Did the participants actively engage in the group activities? | 2.00 (2.00) |
| *Messages* |  |
| 1. Was the number of messages related to interventions appropriate? | 4.00 (0.00) |
| 2. Did the participants carefully read the messages? | 3.00 (1.00) |

Table S4. Characteristics of participants (N = 56)

| Variables | Intervention group (*n* = 27) | Control group (*n* = 29) |
| --- | --- | --- |
| Gender  Male  Female | 13 (48.15)  14 (51.85) | 13 (44.83)  16 (55.17) |
| Grade  1^st^ year  2^nd^ year  3^rd^ year  4^th^ year | 3 (11.11)  5 (18.52)  7 (25.93)  12 (44.44) | 2 (6.90)  5 (17.24)  6 (20.69)  16 (55.17) |
| Major  College of Social Sciences  School of Business | 19 (70.37)  8 (29.63) | 24 (82.76)  5 (17.24) |

Table S5-A. Minimum and maximum scores for all study outcomes across assessment points in the full analytic sample (N = 56)

|  | Intervention Group (*n* = 27) | | | | Control Group (*n* = 29) | | | |
| --- | --- | --- | --- | --- | --- | --- | --- | --- |
| Outcomes | Pre | Mid | Post | 1-month Follow-up | Pre | Mid | Post | 1-month Follow-up |
| Min–Max | | | | | | | | |
| LTPA time | 0–720 | 0–720 | 0–750 | 0–720 | 0–201 | 0–149 | 0–171 | 0–138 |
| Intention | -1.0–4.0 | -2.7–4.0 | -2.0–4.0 |  | -4.0–4.0 | -3.3–4.0 | -3.7–4.0 |  |
| BB | 2.0–4.0 | -3.0–4.0 | 0–4.0 |  | -1.0–4.0 | 0–4.0 | 1.0–4.0 |  |
| OE | 2.0–4.0 | 2.0–4.0 | 3.0–4.0 |  | -1.0–4.0 | -2.0–4.0 | 2.0–4.0 |  |
| NB | 0–4.0 | -1.0–4.0 | 1.0–4.0 |  | -4.0–4.0 | -1.0–4.0 | 0–4.0 |  |
| MC | -3.0–4.0 | -3.0–4.0 | -2.0–4.0 |  | -3.0–4.0 | -3.0–4.0 | -1.0–4.0 |  |
| CB | -3.0–4.0 | -3.0–4.0 | -3.0–4.0 |  | -3.0–4.0 | -3.0–4.0 | -3.0–4.0 |  |
| PP | -4.0–4.0 | -2.0–4.0 | -2.0–4.0 |  | -3.0–4.0 | -4.0–4.0 | -3.0–4.0 |  |

Abbreviation: BB = Behavioral Belief; OE = Outcome Evaluation; NB = Normative Belief; MC = Motivation to Comply; CB = Control Belief; PP = Perceived Power; Min = Minimum score; Max = Maximum score

Table S5-B. Minimum and maximum scores for all study outcomes across assessment points among the first sub-groups (N = 27)

|  | Intervention Group (*n* = 13) | | | | Control Group (*n* = 14) | | | |
| --- | --- | --- | --- | --- | --- | --- | --- | --- |
| Outcomes | Pre | Mid | Post | 1-month Follow-up | Pre | Mid | Post | 1-month Follow-up |
| Min–Max | | | | | | | | |
| LTPA time | 0–120 | 0–450 | 0–750 | 0-600 | 0–120 | 0–270 | 0–420 | 0–240 |
| Intention | -1.0–4.0 | -2.7–4.0 | -2.0–4.0 |  | -4.0–3.33 | -3.0–4.0 | -3.7–4.0 |  |
| BB | 2.0–4.0 | 1.0–4.0 | 2.0–4.0 |  | 2.0–4.0 | 1.0–4.0 | 1.0–4.0 |  |
| OE | 2.0–4.0 | 3.0–4.0 | 3.0–4.0 |  | 3.0–4.0 | 1.0–4.0 | 2.0–4.0 |  |
| NB | 0–4.0 | 1.0–4.0 | 2.0–4.0 |  | -4.0–4.0 | 0–4.0 | 0–4.0 |  |
| MC | -3.0–4.0 | 0–4.0 | -2.0–4.0 |  | -2.0–4.0 | -3.0–4.0 | -1.0–4.0 |  |
| CB | -2.0–4.0 | -2.0–4.0 | -2.0–4.0 |  | -2.0–4.0 | -2.0–4.0 | -2.0–4.0 |  |
| PP | 0–4.0 | -2.0–4.0 | 0–4.0 |  | -2.0–4.0 | -2.0–4.0 | -3.0–4.0 |  |

Abbreviation: BB = Behavioral Belief; OE = Outcome Evaluation; NB = Normative Belief; MC = Motivation to Comply; CB = Control Belief; PP = Perceived Power; Min = Minimum score; Max = Maximum score

Table S5-C. Minimum and maximum scores for all study outcomes across assessment points among the second sub-groups (N = 20)

|  | PHIA Group (*n* = 6) | | | | Control Group (*n* = 14) | | | |
| --- | --- | --- | --- | --- | --- | --- | --- | --- |
| Outcomes | Pre | Mid | Post | 1-month Follow-up | Pre | Mid | Post | 1-month Follow-up |
| Min–Max | | | | | | | | |
| LTPA time | 60–120 | 60–450 | 75–750 | 60–600 | 0–120 | 0–270 | 0–420 | 0–240 |
| Intention | 1.7–4.0 | 2.0–4.0 | 2.0–4.0 |  | -4.0–3.33 | -3.0–4.0 | -3.7–4.0 |  |
| BB | 2.0–4.0 | 2.0–4.0 | 3.0–4.0 |  | 2.0–4.0 | 1.0–4.0 | 1.0–4.0 |  |
| OE | 2.0–4.0 | 3.0–4.0 | 3.0–4.0 |  | 3.0–4.0 | 1.0–4.0 | 2.0–4.0 |  |
| NB | 0–4.0 | 2.0–4.0 | 2.0–4.0 |  | -4.0–4.0 | 0–4.0 | 0–4.0 |  |
| MC | -3.0–4.0 | 1.0–4.0 | 2.0–4.0 |  | -2.0–4.0 | -3.0–4.0 | -1.0–4.0 |  |
| CB | -2.0–4.0 | -2.0–2.0 | -2.0–3.0 |  | -2.0–4.0 | -2.0–4.0 | -2.0–4.0 |  |
| PP | 0–4.0 | -2.0–3.0 | 0–4.0 |  | -2.0–4.0 | -2.0–4.0 | -3.0–4.0 |  |

Abbreviation: BB = Behavioral Belief; OE = Outcome Evaluation; NB = Normative Belief; MC = Motivation to Comply; CB = Control Belief; PP = Perceived Power; Min = Minimum score; Max = Maximum score
